# Exploring the influence of wilderness on conscious awareness

**DOI:** 10.1192/bjo.2021.749

**Published:** 2021-06-18

**Authors:** Oli Purnell

**Affiliations:** Swansea University Medical School

## Abstract

**Aims:**

This study examined what effect wilderness has on our conscious awareness, and by extension of that meta-cognition; our physical, mental and spiritual health.

**Background:**

**Method:**

It reviews available scientific and artistic literature and integrates this with interviews in order to generate original grounded theory.

**Result:**

It was found that intensity of wilderness experience varied proportionally with four mediators; Challenge, Immersion, Beauty and Time. With these maximised, experiences broadly within four themes occurred; Increased Awareness, Confidence, Perspective and Connectedness. When sufficiently intense, these four experiences amalgamated to elicit what Maslow described as 'Peak Experience'.

**Conclusion:**

As such, this thesis unexpectedly provides a pragmatic recipie towards peak experience, and a map of one's potential psychological journey, in the context of wilderness.

